# Incorporation of unfermented or fermented de-oiled rice bran meal into a rabbit’s diet impacts growth performance, nutrient digestibility, cecal microbiota composition, and intestinal barrier function

**DOI:** 10.5713/ab.24.0890

**Published:** 2025-04-11

**Authors:** Shehata Zeid, Sindaye Daniel, Liao Jinghong, Suqin Hang

**Affiliations:** 1 National Center for International Research on Animal Gut Nutrition, Jiangsu Key Laboratory of Gastrointestinal Nutrition and Animal Health, Laboratory of Gastrointestinal Microbiology, Nanjing Agricultural University, Nanjing, China; 2 Department of Animal and Poultry Production, Faculty of Agriculture, Damanhur University, Damanhur, Egypt

**Keywords:** Cecum Microbiota Diversity, Digestibility, Intestinal Health, Performance, Rabbit, Rice Bran Meal

## Abstract

**Objective:**

This study assessed the effects of incorporating unfermented or fermented rice bran meal (RBM) into a rabbit’s diet on their growth performance, cecal microbiota, and intestinal health.

**Methods:**

Twenty-one male weaned New Zealand White rabbits aged 6 weeks were randomly allocated into 3 groups (n = 7). Rabbits consumed the basal diet in the control (CON) group and the basal diet with a 20% substitution of either unfermented RBM (UFRBM) or fermented RBM (FRBM) for 8 weeks. A combination of *Lactobacillus johnsonii* L63 with hydrolytic enzymes ([cellulase (100 U/g], phytase [1.25 U/g], and papain [300 U/g]) was used to FRBM for 60 h at 37°C and a pH value of 4.8.

**Results:**

Our results demonstrated that the rabbits’ growth performance, serum biochemical variables, and cecal microbiota for α and β diversities at the phylum level didn’t differ among the treatments. The nutrient digestibility, cecal and jejunal morphology, or α-amylase and trypsin activities increased in the FRBM group than the CON and UFRBM groups, while the catalase and malondialdehyde activities decreased in the UFRBM group (p<0.05). The solute carrier families 15 and 5 levels were higher in the FRBM group than the UFRBM and CON groups, while the UFRBM group improved the levels of the solute carrier family 1, occludin, and V9D2013 group at the genus level than the FRBM and CON groups (p<0.05). The abundance of glycolysis-to-gluconeogenesis was increased in the FRBM and UFRBM groups compared to the CON group (p<0.05). The total short-chain fatty acid, acetate, and butyrate concentrations were, respectively, improved in the FRBM group than the CON and UFRBM groups (p<0.05).

**Conclusion:**

The two formulas incorporating 20% of UFRBM or FRBM in rabbit diet should be recommended to farmers, particularly the FRBM, to decrease the reliance on corn and soybean meal for rabbit production.

## INTRODUCTION

For many years, corn and soybean meal (SBM) have been relied on as the main feedstuff ingredients that provide a suitable protein and energy source for animals [1–3]. The high demand for these feedstuffs’ materials led researchers to seek alternatives, aiming to reduce the dependence on them [4,5]. Among these alternatives, de-oiled rice bran meal (RBM) has been proposed [6–8]. RBM is a sufficient source of nutrients such as protein (15.0% to 17.0%), crude fiber (13.0% to 17.0%), lipids (1.2% to 2.7%), crude ash (8.0% to 13.0%) and nitrogen free extract (59.1% to 66.0%) required by animals [9–11]. The anti-nutritional factors (ANFs), such as high content of fiber and phytate in RBM, make it less useful in animal production and particularly monogastric animals [12,13]. The New Zealand rabbit, a typical meat producer, can tolerate the high level of crude fiber and may valorize RBM without more difficulties [14]. Appropriate technique can reduce the ANFs content in RBM [15], and solid-state fermentation (SSF) has been adopted as a useful technique [16,17]. Some reports have shown that RBM fermentation by beneficial bacteria could improve its nutritive value [18,19].

The findings cleared that feeding pigs a diet incorporating 10% of de-oiled RBM fermented by a mixture of *Lactobacillus plantarum*, *Bacillus subtilis*, *Saccharomyces cerevisiae*, and phytase enzyme could improve their growth performance, gastrointestinal health, intestinal microbiome, nutrient digestibility, and antioxidant parameters [20]. Feeding crap fish (Labeo rohita) a diet containing 90% of defatted RBM supplemented with microbial phytase and xylanase could enhance their growth performance and nutrient utilization [21]. In an *in vitro* study, it was shown that fermentation of de-oiled RBM by *Rhizopus oryzae* could enhance the n-6 fatty acid profile and decrease the phytate and trypsin inhibitor content [13]. Another *in vitro* study demonstrated that fermented defatted RBM by *Bacillus subtilis* could increase the phenolic compounds content and enhance the antioxidant capacity [22]. Laying hens receiving a diet supplemented with two levels (2.5% and 5%) of heat-treated rice bran (RB) fermented by *Bacillus subtilis* and *Lactobacillus plantarum* improved the production performance, nutrients digestibility, and altered the cecal microbiota linked to short-chain fatty acid (SCFA) production, with optimal results observed at the 5% level [23]. The mice given a high-sucrose diet with 20% RB fermented by yeast (*Saccharomyces cerevisiae*) and bacteria (*Lactobacillus plantarum*) showed improvements in their blood lipid state and intestinal health after the fermentation [24]. There were limited studies on the effects of feeding rabbits with a diet containing fermented RBM (FRBM) treated by different cellulolytic bacteria and hydrolytic enzymes. It has been proved that lactic acid bacteria combined with hydrolytic enzymes decreased the ANFs and improved the nutritional value of RBM through the fermentation process [25,26].

Among the beneficial lactic bacteria, *Lactobacillus johnsonii* attracted our attention for the feed industry. However, *Lactobacillus johnsonii* L63 (*L. johnsonii* L63), a new strain of bacteria isolated and preserved in our laboratory (laboratory of gastrointestinal microbiology at Nanjing Agricultural University, Nanjing, China), combined with hydrolyzed enzymes, was used in this study to ferment the RBM. Our previous studies in our laboratory in *in vitro* experiments, the fermentation of RBM with *L. johnsonii* L63 combined with cellulase, phytase, and papain enzymes showed an improvement in the RBM nutrients value [27], and it’s functionality of antioxidant capacity and host intestinal health protection [28].

The purpose of this study was to evaluate the substitution of corn and SBM in rabbit diets by either unfermented RBM (UFRBM) or RBM fermented by a combination of *L. johnsonii* L63 and the hydrolytic enzymes (FRBM) on their productivity. This study revealed that *L. johnsonii* L63 combined with hydrolysis enzymes improved the nutritional value of defatted RBM during the fermentation process. Feeding rabbits a diet replacing corn and SBM with FRBM improves the nutrient digestibility, intestinal morphology, antioxidant capacity, digestive enzymes, nutrient transporter expression, SCFA concentration, the abundance of glycolysis-to-gluconeogenesis and carbohydrate digestion and absorption metabolic pathways. This study provides two formulas (FRBM or UFRBM) that can decrease the dependence of corn and SBM and offers new insights into rabbit production.

## MATERIALS AND METHODS

### Animal care ethics

The protocols and methodologies used in our study were approved and authorized by the Animal Welfare and Ethics Committee of Nanjing Agricultural University, Nanjing, China (License: NJAU No. 20230427070). This study was supported by the National Key R&D Program Projects, 14th Five Year, grant No: 2021YFD1300301-5 (2021 to 2026).

### Animals, experimental design, diets, and housing

A total of 21 male weaned New Zealand White rabbits aged 6 weeks with a similar body weight (1,287±110.86 g) were purchased from Laifu Farm, Pukou District, Nanjing, China. All rabbits were randomly distributed into 3 groups (n = 7), namely, the control (CON) group, the UFRBM group, and the FRBM group. Each group had seven replicates, with one rabbit in each replicate. The rabbits consumed a basal diet in the CON group, whereas in the UFRBM or FRBM groups, rabbits received a diet substituted by either 20% of UFRBM or FRBM (Supplement 1). In *in vitro* studies, RBM fermentation showed a reduction in the crude fiber and ANFs, resulting in the improvement of nutrients bioavailability [28]. As the rabbits can tolerate high crude fiber content in their diet [29], they received 20% of FRBM in the FRBM group and 20% of UFRBM as a negative control in the UFRBM group. Since the price of RBM was lower than that of corn and SBM, the cost of the UFRBM diet was lower than that of the CON, while the cost of FRBM nearly equaled that of the CON due to the costs associated with the fermentation process (Supplement 1). All diets were formulated to meet the growing rabbit’s nutritional requirements according to the Chinese Agricultural Standard Publishing Research Center (Issue No. NY/T4049-201).The experiment ran for eight weeks (one week for adaptation and seven weeks for growing). Rabbits were grown under the same hygienic conditions in automated controlled housing, maintaining a 16 to 8 h light-dark cycle and 25°C of temperature throughout the entire experiment period. Each rabbit was separately housed in an individual cage (60×50×40 cm) and provided with unlimited access to fresh water and the mash diet ad libitum. Because *L. johnsonii* L63, which was used during the RBM fermentation process, was not resistant to high temperatures (the temperature required for manufacturing the pellet feed), the mixing feed was done manually every day, and the feed was presented to rabbits as a mash feed diet.

### Rice bran meal fermentation

*Lactobacillus johnsonii* L63 was isolated, identified, and characterized in the laboratory of the gastrointestinal microbiome at Nanjing Agricultural University, Nanjing, China [27]. For this experiment, 1% of *L. johnsonii* L63 was mixed with 1% De-Man-Rogosa-Sharpe Broth (a culture medium that contains sodium acetate for growing l*actobacillus* species) before being dissolved in 1,000 mL. The bacterial solution was inoculated at 2% of the total solution volume (v:v). During this research, cellulase, phytase, and papain enzymes with 100, 1.25, and 300 U/g enzyme activities, respectively, were hydrolyzed in the same water and poured into the prepared solution containing *L. johnsonii* L6*3*. The obtained mixture solution was mixed with RBM in a 1:1 v/w ratio before being packed in plastic bags with a one-way valve prepared specifically for fermentation. The packed RBM was fermented in the incubator for 60 h at 37°C and a pH of 4.8. After the SSF stage was finished, the FRBM was stored at room temperature. The Supplementa 2 presents the nutritional value of UFRBM and FRBM.

### The growth performance evaluation

After one week of adaptation, the initial body weight (IBW) was recorded with no notable variation between the groups. During the rabbit growing phase, the body weight gain was determined weekly, and the feed intake was calculated every day. At the end of the experimental time, the final body weight (FBW) was recorded. The following equations were used for determining the average daily gain (ADG), average daily feed intake (ADFI), or gain-to-feed ratio as follows: ADG = (FBW−IBW)/number of the trial days; ADFI = total feed intake/number of the trial days; and gain-to-feed ratio = ADG/ADFI.

### Apparent nutrient digestibility determination

On the last three experimental days, the feces samples were collected manually from each rabbit (n = 7 rabbits per group). For fixing the excreta nitrogen, HCl (10%) was added before being kept at −20°C for digestibility analysis. The representative feed and feces samples were dried in the oven at 60°C for 48 h, ground until they could pass through a 1 mm screen, packed in specific plastic bags, and stored at 4°C until digestibility determination. The digestibility of dry matter (934.01, AOAC), gross energy (IKA calorimetric system C5010, Staufen, Germany), crude protein (988.05 AOAC), ether extract (GB/T6433-2006), acid-insoluble ash (942.05 AOAC), and crude fiber, neutral detergent fiber, or acid detergent fiber (ANKOM A200i method) were tested following the manufacturer’s instructions for the digestibility standard method determinations. The next equation was used to calculate the apparent nutrient digestibility:


$$Nitrients\;Digestabelity=100\times \left[1-\left(\frac{(AIA)\;diet}{(AIA)\;feces}\right)\times \left(\frac{(\text{N})\;feces}{(N)\;diet}\right)\right]$$

the term AIA means the acid-insoluble ash amount, while N signifies the fecal and feed nutrient concentration.

### Serum biochemical variables analysis

After the experiment was finished, 5 mL of blood was collected for each fasted rabbit before being manually slaughtered following the routine bleeding method by cutting the trachea, esophagus, carotid artery, jugular vein, and vagus nerve. The collected blood samples were kept at room temperature on an inclined plate until the serum became precipitated, then centrifuged at 3,000×g at 4°C for 10 min. The samples of serum obtained were stored at −80°C for the analysis of biochemical variables. The determinations of serum glucose, triglycerides, total cholesterol, total protein, globulin, albumin, creatinine, blood urea nitrogen, alkaline phosphatase, alanine aminotransferase, and aspartate aminotransferase levels were tested by using an automatic biochemical analyzer system (Chemray 800; Shenzhen Rayto Life Science Co., Ltd., Shenzhen, China).

### Antioxidant and immunity indices examination

The purpose of the antioxidant and immunity variable detection in this experiment was to prove evidence that the *L. johnsonii* L63 bacteria used in the fermentation process didn’t have any effects on the rabbit’s health and the oxidative status. The jejunal mucosa tissue was scraped, weighed (0.1 g), mixed with 0.9 mL of physiological saline, homogenized, and centrifuged. To determine the antioxidants and immunity parameters, the supernatant was collected and kept. The concentration of protein used for calculating the antioxidant and immunity parameters in the mucosal tissue samples was analyzed by a bicinchoninic acid protein assay kit (P0006C method). The activity of antioxidant indices, including total antioxidant capacity (A015-2-1 method), superoxide dismutase (A001-3 method), glutathione peroxidase (A005 method), catalase activity (A007 method), and the lipid peroxidation of malonaldehyde level (A003-1 method), was measured to evaluate the anti-oxidative status.

For the immunity parameter, 0.1 g of jejunal mucosa samples were mixed with 900 μL of pre-cooled phosphate buffered saline (pH 7.4) in a 2 mL centrifuge tube, homogenized, and then centrifuged at 2,500×g for 20 min at 4°C. The supernatant obtained was collected to determine the secretory immunoglobulin A (sIgA) level using an enzyme-linked immunosorbent assay kit specifically for rabbits. All antioxidant and immunity parameters or tissue protein concentration measurements followed the instructions given by the manufacturer for the commercial kits (Nanjing Jiancheng Bioengineering Institute, Nanjing, China).

### Intestinal histomorphology measurement

The jejunal and cecal tissue samples were cut from the tissue midpoint and fixed instantly for 24 hours in 4% paraformaldehyde for histological examination. The tissue samples were sliced into pieces and put in a 10% neutral formalin fixative and then manually dehydrated. The samples were drenched and kept in paraffin wax before being cut into 5 μm slices, and the stored samples were stained with hematoxylin and eosin. A digital camera installed on a microscope was used to capture the histological images (Olympus BX5; Olympus Optical Co. Ltd., Tokyo, Japan). The histological indices, such as villus height (VH) and crypt depth (CD), were examined by using the Image Pro Plus software (version 6.0; Media Cybernetics, Inc., Rockville, MD, USA). At least eight well-cleared villi per section must be captured for the intestinal histomorphology measurement.

### Digestive enzyme activity determination

A total of 0.1 g of jejunal digesta samples were mixed with 0.9 ml of saline solution until they became mixed. The solution obtained was homogenized and centrifuged at 8,000 ×g at 4°C for 10 min. The collected supernatant was used to test the digestive enzyme activity. Following the instructions designed by the manufacturer (Nanjing Jiancheng Bioengineering Institute, Nanjing, China), the commercial assay kits were used to test the enzyme activities of α-amylase (C016-1-1 method), lactase (A082-3-1 method), lipase (A054-2-1 method), trypsin (A080-2 method), and chymotrypsin (A080-3-1 method).

### RNA extraction and quantitative real-time polymerase chain reaction

According to manufacturer instructions, TRIZOL reagent (AG21024; Accurate Biotechnology Co., Ltd., Hunan, China) was used to extract the jejunal mucosal ribonucleic acid (RNA). A Nano-Drop 2000 spectrophotometer (Thermo Scientific, Waltham, MA, USA) measured RNA content and purity from OD260/280 readings (ratio >1.8). The total RNA was converted into cDNA by using the Quantitect Reverse Transcription Kit (AG11728; Accurate Biotechnology Co., Ltd.). The mRNA gene expression levels of targeted genes for the nutrient transporters and the tight junctions were tested by using the Bio-Rad CFX96 real-time PCR system (Bio-Rad Laboratories, Hercules, CA, USA). The normalization against the housekeeping gene was done before the mRNA relative expression levels of targeted genes were assessed by the 2−ΔΔCt approach. As shown in Supplement 3, all of the primers detected in this research were designed for *Oryctolagus cuniculus* by using NCBI-Primer BLAST (NCBI Taxonomy ID 9986) or Sangon Biotechnology (Sangon Biotech, Shanghai, Co., Ltd., China), and synthesized by Invitrogen Life Technologies (Shanghai, China).

### Short-chain fatty acid analysis

Capillary column gas chromatography (GC-14B; Shimadzu, Kyoto, Japan; capillary column: 30 m×0.25 mm×0.25 μm, film thickness) was used to test the cecal digesta SCFA concentration. In short, 0.5 g of cecal digesta samples were dissolved in 1,000 μL of ddH_2_O and then centrifuged at 12,000×g for 10 min in a microcentrifuge (Microfuge 22R; Beckman Coulter, Brea, CA, USA). The collected supernatant was mixed with 100 μL of crotonic acid (as an internal standard for SCFAs analysis). A flame ionization detector and a capillary column (30 m×0.25 mm×0.25 μm, HP-INNOWax; Agilent Technologies, Santa Clara, CA, USA) were used to test the SCFA. The injector and detector were heated to 180°C, while the column was kept at 130°C with a gas flow rate of 30 mL/min.

### 16S rRNA microbiota sequences analysis

Following the instructions given by the manufacturer, a DNA extraction kit (Omega Bio-Tek, Norcross, GA, USA) was used to extract the pure genomic DNA from 0.3 g of cecal digesta. Primers 341F and 806R were used to amplify the 16S rRNA gene’s V3–V4 region. After that, sequencing was produced using the pooled DNA product in accordance with Illumina’s protocol. The Illumina MiSeq platform was then used to perform paired-end sequencing on the library. To find sequences, UCHIME was used. At 97% similarity, the sequences were grouped together as a single operational taxonomic unit (OTU). Alpha diversity was performed by refining the OTU abundance data. The Phylogenetic Investigation of Communities by Reconstruction of Unobserved States tool, which is based on the Kyoto Encyclopedia of Genes (KEGG) and Genomes database, was used to predict different samples’ functional changes in microbiota.

### Statistical analysis

The experiment used an entirely random design, and the statistical analysis was analyzed by SPSS 25.0 software (SPSS Inc., Chicago, IL, USA). All data analysis was done using a one-way ANOVA with a linear polynomial model. The significant differences between the treatments were performed using the Tukey test for the multiple comparisons of the means. The results were tabled as mean and pooled standard error of mean, and a p-value less than 0.05 was required for significance assertions. Based on unweighted UniFrac distance metrics, the principal component analysis (PCA) and principal coordinate analysis (PCoA) were used to compare groups of samples.

## RESULTS

### Growth performance

As shown in Table 1, the UFRBM and FRBM groups didn’t vary from the CON group in terms of FBW, ADG, and gain-to-feed ratio. The ADFI was considerably higher in the FRBM and UFRBM groups than that in the CON group (p<0.05), but there was no big difference observed across the UFRBM and FRBM groups.

### Apparent nutrient digestibility

As presented in Table 2, there were no recognized variations in terms of apparent digestibility of dry matter, organic matter, neutral detergent fiber, and acid detergent fiber across FRBM and UFRBM groups or when each treatment was compared to the CON group. In comparison to the UFRBM or CON groups, the FRBM group improved the digestibility of gross energy, crude protein, and crude fiber (p<0.05), but those in the UFRBM group did not vary from those in the CON groups. The digestibility of ether extract was higher in the FRBM group compared to that in the UFRBM group (p<0.05), and it was improved in both of these 2 groups compared to that in the CON group (p<0.05). There was an increase (p<0.05) in crude ash digestibility in the UFRBM and FRBM groups relative to the CON group, but no difference was observed between the UFRBM and FRBM groups.

### Serum biochemical variables

The results presented in Table 3 revealed that there was no difference for all serum biochemical variables, including blood urea nitrogen, glucose, triglycerides, total cholesterol, total protein, globulin, albumin, creatinine, aspartate aminotransferase, alkaline phosphatase, and alanine aminotransferase across the FRBM and UFRBM groups or when each treatment was compared to the CON group.

### Antioxidant indices and immunity status

As indicated in Table 4, there was no discernible change in catalase activity level across the FRBM and CON groups, whereas the FRBM group’s catalase activity went up (p<0.05) more than the UFRBM group. The UFRBM group exhibited a lower malondialdehyde concentration than the FRBM and CON groups, respectively (p<0.05). However, no big differences were observed between the FRBM and CON groups. Regarding other antioxidant indices, there were no differences between the treatments and the CON group in terms of total antioxidant capacity, glutathione peroxidase, superoxide dismutase, or sIgA.

### Intestinal histomorphology

**Figure 1:**
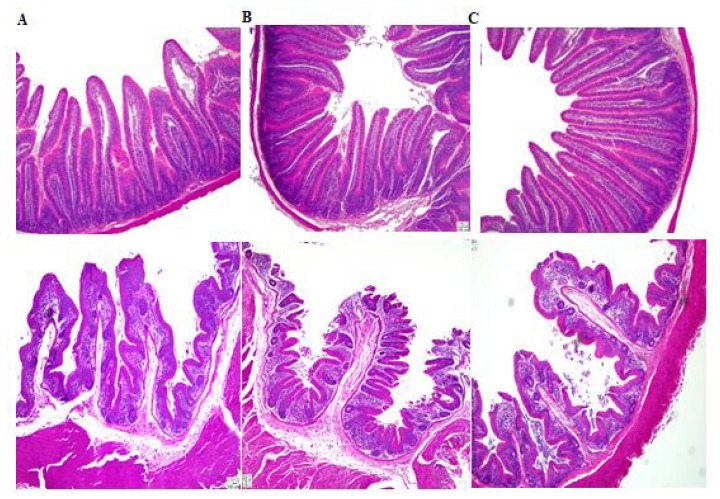
The intestinal histological appearance in the jejunum and cecum of rabbits; (A) CON rabbits for jejunum and cecum (B) UFRBM rabbits for jejunum and cecum and (C) FRBM rabbits for jejunum and cecum (Scale bars = 100 μm). CON, control group; UFRBM, unfermented rice bran meal group; FRBM, fermented rice bran meal group.

As shown in Table 5 and Figure 1, the VH in the jejunum and cecum, the VH:CD of the jejunum and cecum, and the jejunal CD in the FRBM group were better than those in the UFRBM and CON groups (p<0.05). Furthermore, the jejunal VH in the UFRBM group was substantially longer than that in the CON group (p<0.05). There was no change in the cecal CD observed among the treatments. There were no big variations found in jejunal and cecal VH:CD or jejunal CD between the UFRBM and CON groups.

### Digestive enzyme activity

Table 6 indicates that the α-amylase activity in the FRBM group was substantially higher than the UFRBM group (p<0.05). However, no noted variation was observed among the FRBM and CON groups or the UFRBM and CON groups. Trypsin activity was considerably higher in the FRBM group than in the CON group (p<0.05). However, there were no changes noted between the FRBM and UFRBM groups or the UFRBM and CON groups. On the other hand, the data showed that lactase, lipase, and chymotrypsin activities weren’t different in all treatments and the CON group.

### The mRNA expression of jejunal nutrient transporters and tight junction genes

The results of the gene expression for nutrient transporters and tight junctions are presented in Table 7. The FRBM upregulated the *SLC15A1* and *SLC5A1* mRNA expression in comparison to those in both the UFRBM and CON groups (p<0.05), but no regulation changes were recognized among the UFRBM group and CON group. The FRBM downregulated the *SLC5A10* compared to that in the CON group (p<0.05), but no big changes were found among the UFRBM and CON groups or the UFRBM and FRBM groups. While there was no difference in *SLC3A1* expression between the UFRBM and FRBM groups or the FRBM and CON groups, the UFRBM group elevated the expression of *SLC3A1* and *SLC1A1* when compared to those in the CON group (p<0.05). The expression of *SLC1A1* was raised (p<0.05) in the UFRBM compared to that in the FRBM. There was no big change in *FABP1* expression among the FRBM and UFRBM groups or when each treatment was compared to the CON group. In the UFRBM group, the expression of *OCLN* was enhanced more than in the FRBM or CON groups (p<0.05). However, there was no change in *OCLN* expression in the FRBM or CON groups. All treatments didn’t show any regulation in *ZO-1* and *CLDN1* expression compared to those in the CON group.

### Cecum microbiota

**Figure 2:**
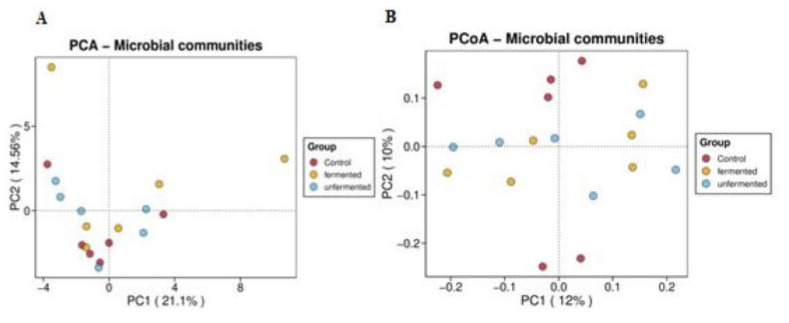
(A) Principal component analysis distribution and (B) principal coordinate analysis plot of rabbit’s cecum bacterial community.

**Figure 3:**
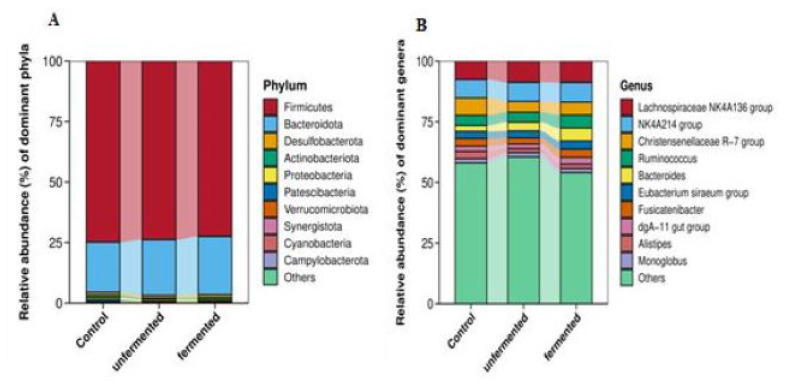
The relative abundance of dominant cecum microbiota composition at phylum (A) and genus (B) levels of New Zealand White rabbits.

There were no recognized changes found in the α-diversity of the microbiota for richness and diversity indexes among the treatments (Table 8). The PCA and the PCoA of the cecum bacterial community presented in Figures 2A, 2B) revealed that the rabbits fed the basal diet were not separated from those consuming the UFRBM or FRBM, with the 2 principal coordinates (PC1 and PC2) explaining, respectively, 21.1% and 14.6% and 12% and 10%. The relative abundance of the cecum microbiota at phylum and genus levels was described in Figures 3A, 3B). As illustrated by Figure 3A, the dominant phyla were the *Firmicutes* and *Bacteroidota*, while at the genus level (Figure 3B), the *Lachnospiraceae NK4A136 group*, *NK4A214 group*, *Christensenellaceae R-7 group*, *Ruminococcus*, *Bacteroides*, *Eubacterium siraeum group*, and *Fusicatenibacte*r dominated the rabbit’s cecum microbiota. As shown in Table 9, the top 30 dominant genera indicated that the FRBM group decreased the relative abundance of *Rikenella* and *Anaerostipes* than UFRBM group (p<0.05), while the UFRBM group increased the relative abundance of the *V9D2013 group* and *Rikenella* than the CON group (p<0.05). However, there were no differences in the relative abundance of the *V9D2013 group* between the UFRBM and FRBM groups or the FRBM and CON groups. The relative abundance of *Anaerostipes* did not differ across the UFRBM and CON groups or the FRBM and CON groups. There was no difference in the relative abundance of *Rikenella* between the FRBM and CON groups. Furthermore, no big change was observed in the remaining top 30 genera among the treatments.

In this study, the functional predictions of metabolic pathways for the cecal microbiota were evaluated by the Kyoto Encyclopedia of Genes and Genomics at 3 levels (level 1, level 2, and level 3). The KEGG metabolic pathways of cecum microbiota at level 1 (Supplement 4) were categorized into 6 categories; among them, the metabolic pathways associated with the metabolism were predominant in all treatments (more than 75%). At KEGG level 2, the metabolic pathways related to the metabolism showed that the carbohydrate metabolism and amino acid metabolism were predominant in all treatments (Supplement 5). The results indicated that there was no noteworthy change observed in the KEGG metabolic pathways for the cecal microbiota at levels 1 and 2 among the treatments. As shown in Table 10, at KEGG level 3, the functional predictions of carbohydrates indicated that the FRBM and UFRBM groups improved the metabolic pathways of glycolysis-to-gluconeogenesis more than the CON group (p<0.05), but there was no big difference between the FRBM and UFRBM groups. The FRBM group enhanced the metabolic pathways of carbohydrate digestion and absorption more than the UFRBM or CON groups (p<0.05), but there was no change noted between the UFRBM and CON groups. The FRBM group decreased the butanoate metabolism compared to that in the UFRBM group (p<0.05), but it didn’t differ across the FRBM and CON groups. However, there were no differences in the remaining detected carbohydrate metabolic pathways.

### Short-chain fatty acid concentration

As shown in Table 11, the FRBM group enhanced the total SCFAs and acetic acid concentrations more than those in the CON group (p<0.05), while there wasn’t a big difference among the FRBM and UFRBM groups or the UFRBM and CON groups. An increase was found for butyric acid concentration in the FRBM group than the UFRBM group (p<0.05), but no difference was recognized between the CON and FRBM groups or CON and UFRBM groups. All treatments didn’t show any noted changes in propionic, isobutyric, valeric, and isovaleric acid concentrations compared to those in the CON group.

## DISCUSSION

### Growth performance

The growth performance of rabbits is an important factor that directly affects the economy of farmer. No differences were observed in the FBW, ADG, and gain-to-feed ratio across all groups, indicating that appropriate substitution of UFRBM or FRBM in rabbit diets didn’t cause any side effects on their growth. A higher ADFI was found in the FRBM and UFRBM groups than that in the CON group, which could be explained by the different sources of nutrients provided during our feed formulation, particularly the composition of UFRBM or FRBM compared to corn and SBM (energy and crude protein content). Despite the lack of evidence variations in rabbits’ FBW among the groups, the FRBM group showed a numerical increase in FBW (2,813.33±50.39 g) more than the CON group (2,600.00±125.47 g), which justified the greatest digestibility, intestinal morphology, nutrient transporters, digestive enzyme activity, and SCFA found in the FRBM group.

### The apparent nutrient digestibility

Our experiment revealed that the digestibility of gross energy, crude protein, crude fiber, and ether extract in the FRBM group was much improved in comparison to that in the UFRBM and CON groups, which might result in the decreased ANFs, crude fiber content, and the enhancement of nutrient bioavailability during the RBM fermentation process. It provided soluble carbohydrates, amino acids, and elements for absorption in the intestinal tract. The increase in the digestibility of ether extract and crude ash in the UFRBM group in comparison to those in the CON group was influenced by the RBM’s nutritional composition. There is a similarity between our finding and the previous studies, which demonstrated that RBM fermentation with a combination of cellulolytic bacteria and hydrolytic enzymes could improve the apparent nutrient digestibility of pigs [20] and laying hens [23].

### The serum biochemical variables

Results of this study showed that there were no noticeable differences in the serum biochemical variables between the treatments (FRBM and UFRBM) and the control group. Despite the lack of evidence in the biochemical variables tested in this study, their values stayed in the normal range for rabbits in good health, indicating that rabbits fed a diet incorporating UFRBM and FRBM are not exposed to the risk of diseases. This suggests that *L. johnsonii L63* (combined with hydrolysis enzymes) is safe for use in rabbit feed manufacturing without any side effects on their health.

### Antioxidant indices and immunity status

Our experiment revealed a decrease in malondialdehyde activity in the UFRBM group in comparison to the CON or FRBM groups, indicating that there was an improvement in the anti-oxidative status by preventing lipid peroxidation in the UFRBM group. However, the catalase activity was higher in the FRBM group than that in the UFRBM group, which showed that fermentation of RBM could improve the antioxidant capacity of rabbits by breaking down the ANFs and crude fiber and enhance the bioavailability and release of phenolic antioxidant-rich compounds (such as ferulic acid, γ-oryzanol, and tocopherols) to maximize the protective effect versus damage caused by oxidation and free radicals from the body [22].

In rabbits, the role of mucosal sIgA is crucial, as it protects rabbits from adhering pathogens, infections, and enteric toxins to the gastrointestinal barrier [30]. Our finding revealed that no differences were noted in jejunal mucosal sIgA among treatments, indicating a better immune status in rabbits [31].

### Intestinal histomorphology

Our findings showed an increase in the jejunal and cecal VH, CD, and VH:CD in the FRBM than the CON and UFRBM groups, indicating a better improvement in the rabbit intestine’s structure and an increase in its capacity to absorb nutrients. The VH was improved in the UFRBM group more than the CON group, showing that feeding rabbits with a diet incorporating UFRBM could improve the area of nutrient absorption. As well as the fact that rabbits tolerate a high amount of fiber in their diet, the higher fiber content in the UFRBM may influence the development of rabbit intestinal structure [32].

### Digestive enzyme activity

The digestive enzymes have a direct effect on rabbits’s digestion and absorption of nutrients [33]. Our research revealed that the FRBM group exhibited higher levels of α-amylase enzyme activity than the UFRBM group and trypsin enzyme activity than the CON group. As α-amylase breaks down the complicated starch to simple sugars and trypsin degrades the complex protein into peptides and basic amino acids, the highest digestive enzyme activity of trypsin and α-amylase found in the FRBM group showed that during the fermentation of RBM using a combination of *L. johnsonii L63* and hydrolysis enzymes, it could decrease the crude fiber content and increase the bioavailability of soluble carbohydrates and free amino acids, which are easy to be digested by rabbits. This observation is supported by the highest digestibility of crude protein and gross energy found in the FRBM group.

Furthermore, lipase breaks down lipids to fatty acids, lactase converts lactose to galactose and glucose, while chymotrypsin breaks down peptide bonds containing aromatic amino acids like phenylalanine, tyrosine, and tryptophan [34]. Our findings showed no difference among the treatments in lipase, lactase, and chymotrypsin activities, indicating that the conversion of lipids to fatty acids, lactose to simple sugars, and peptides to aromatic amino acids wasn’t affected by the dietary treatments.

### The mRNA expressions of nutrient transporters and tight junction genes

In the rabbit intestine, the nutrient transporters play sensitive roles in the assimilation of nutrients and can influence growth performance and productivity [30,35]. Our results showed that there was a upregulation in the gene expression of *SLC5A1* and *SLC15A1* in the FRBM group compared to the UFRBM and CON groups, indicating that the RBM fermentation using *L. johnsonii* L63 and hydrolytic enzymes could improve the assimilation of oligopeptides (*SLC15A1*), which account for about 70% of the total nitrogen digested in the body for absorption [36], and glucose (*SLC5A1*), which means that our RBM fermentation strengthened the positive relation of nutrient transporters functionality and digestive enzymes secretion enhancements and increased the ability to absorb nutrients [36,37]. The UFRBM upregulated the expression of *SLC1A1* than the CON and FRBM groups and *SLC3A1* compared to that in the CON group. This remark showed that the UFRBM could enhance the transportation of glutamate (*SLC1A1*) and excitatory amino acids (*SLC3A1*). The mRNA gene expression of *SLC5A10* was downregulated in the FRBM group than the control group, which demonstrated that through the RBM fermentation, the capacity to transport mannose and fructose decreased.

In the rabbit intestine, the role of the tight junction is crucial in maintaining and regulating the barrier function and permeability of nutrients [30,38]. The *OCLN* mRNA expression was higher in the UFRBM group than the CON and FRBM groups. However, there was no regulation found between the CON and FRBM groups. This suggests that adding UFRBM to a rabbit’s diet could improve the function of the optional barrier and tight junction stabilization. Incorporating UFRBM or FRBM into the rabbit’s diet did not affect intestinal barrier function, as demonstrated by the lack of observed expressions of *ZO-1* and *CLDN1* among the FRBM and UFRBM groups in comparison to those in the CON group.

### Cecum microbiota

In rabbits, the cecal microbiota has a vital role in improving their growth performance, feed efficiency, metabolism, and intestinal health [39,40]. Our finding showed that the alpha diversity indices, including observed species reads, Chao, Ace, Shannon, and Simpson, didn’t differ among the FRBM, UFRBM, and CON groups, indicating that feeding rabbits a diet incorporating UFRBM or FRBM did not affect the number of cecal bacteria populations and the richest factor. At beta diversity, our findings revealed that the predominant phyla were *Firmicutes* and *Bacteroidota*, suggesting that they serve as a good source of energy metabolites, promote gut health, and maintain intestinal homeostasis, a finding that aligns with previous studies conducted on chickens [41]. At the genus level, our finding demonstrated that the *Lachnospiraceae NK4A136 group*, *NK4A214 group*, *Christensenellaceae R-7 group*, *Ruminococcus*, *Bacteroides*, *Eubacterium siraeum group*, *Fusicatenibacte*r, *dgA-11 gut group*, and *Alistipes* were predominant bacterial populations. Our finding showed that among the top 30 genera, the relative abundance of the *V9D2013 group* and *Rikenella* was increased in the UFRBM groups than in the CON group, while the relative abundance of *Rikenella* and *Anaerostipes* was decreased in the FRBM group than the UFRBM group. These differences in relative abundance of bacteria at the genus level could be explained by the different sources of fiber provided to the rabbit’s diet during our feed formulation, and the activity of the bacteria involved in fiber degradation might be different. Overall, our diets didn’t have big changes to the cecal microbiota, indicating that the RBM incorporation kept the microbiota diversity and richness balanced as the same in the CON group.

At KEGG pathway level 3, the FRBM group showed a greater increase in the relative abundance of metabolic pathways related to glycolysis-to-gluconeogenesis compared to the CON group, as well as carbohydrate digestion and absorption. This suggests that feeding rabbits FRBM could effectively convert energy metabolites from the cecum to epithelial cells [17]. The UFRBM improved the metabolic pathways of glycolysis-to-gluconeogenesis more than the CON group, indicating that the activity of cecal microbiota involved in crude fiber degradation was more active.

### Short-chain fatty acid concentration

By improving the energy source’s support, nutrient digestibility, gut health, and homeostasis, or immune status, SCFA play a vital role in rabbit performance [42]. Our finding showed that the FRBM group improved the total SCFA and acetic acid concentrations more than the CON group. This might be explained by the activity of microbiota involved in the crude fiber degradation in the rabbit’s cecum, resulting in the highest nutrient digestibility, especially the crude fiber, which is found in the FRBM group. Furthermore, the fermentation of RBM increased the bioavailability of nutrients and provided a beneficial source of energy for the cecum microbiota [20,28,43]. It has been reported that the interaction between gut microbiota activity and the diet that has many types of complex carbohydrates maximizes the production of SCFA content in rabbits [44]. This observation aligns with our own findings, as we used a different source of fiber in our feed formulation. The decrease of butyric acid in the UFRBM group than FRBM group might be due to the high fiber content in the UFRBM [45]. Additionally, during our fermentation process, the activity of *L. johnsonii* L63 and hydrolysis enzymes degraded the crude fiber in RBM, resulting in lower fiber content in the FRBM diet. This provides a suitable source, type, and amount of crude fiber fraction for the cecal microbiota, which produces butyric acid.

Focusing on our results, throughout the fermentation process, *L. johnsonii* L63 combined with hydrolysis enzymes improved the RBM’s nutritional value by degrading the high crude fiber content and decreasing the ANFs. When we compare the findings among the treatments, FRBM outperformed the CON group in the most parameters that were tested in this study and showed a better improvement than the UFRBM. The no resistance to high temperature of *L. johnsonii* L63 limited the manufacture of pellet feed incorporating FRBM, which is a suitable form for rabbit feed.

Based on our findings (high performance, digestibility, intestinal morphology, nutrient transporters, digestive enzyme activity, microbiota diversity, and SCFA) and the cost of the different feed formulae established, the UFRBM and FRBM could be a better alternative to the shortage of corn and SBM in rabbit production.

## CONCLUSION

Feeding rabbits a diet containing 20% of either UFRBM or FRBM did not affect their growth performance, biochemical variables, and immune status. FRBM improved the nutrient digestibility, antioxidant capacity, intestinal histology, digestive enzymes, gene expression of oligopeptides and glucose (*SLC5A1* and *SLC15A1*), SCFA production, and metabolic pathways abundance of glycolysis-to-gluconeogenesis and carbohydrate digestion and absorption, while UFRBM could enhance the nutrient digestibility (ether extract and crude ash), jejunal VH, gene expression of glutamate, excitatory amino acids (*SLC1A1* and *SLC3A1*), tight junction stabilization (*OCLN*), relative abundance of *V9D2013 group* and *Rikenella* at the genus level, and metabolic pathways abundance of glycolysis-to-gluconeogenesis and lowered the malondialdehyde concentration more than the CON group. FRBM improved the digestibility, intestinal histomorphology, and most terms of antioxidant indices, nutrient transporters, butyrate concentration, or carbohydrate digestion and absorption metabolic pathways than the UFRBM. The two formulas proposed for rabbit feeding should be suggested to farmers, particularly the FRBM diet, to reduce their reliance on corn and SBM.

## Figures and Tables

**Table 1:** Effects of incorporation of UFRBM or FRBM into rabbit’s diet on growth performance^1)^

Item	CON	UFRBM	FRBM	SEM	p-value
IBW (g)	1,287	1,277	1,296	26.1	0.962
FBW (g)	2,600	2,753	2,813	50.4	0.212
ADFI (g/d)	114^b^	127^a^	130^a^	2.70	0.039
ADG (g/d)	26.7	30.1	30.9	1.00	0.207
Gain-to-feed ratio	0.231	0.237	0.237	0.0050	0.882

1) n = 7/treatment.

a,b Means with different superscripts in the same row are significantly different (p<0.05).

CON, control group; UFRBM, unfermented rice bran meal group; FRBM, fermented rice bran meal group; SEM, standard error of the mean; IBW, initial body weight; FBW, final body weight; ADFI, average daily feed intake; ADG, average daily gain.

**Table 2:** Impacts of incorporation of UFRBM or FRBM into rabbit’s diet on apparent nutrient digestibility^1)^

Item (%)	CON	UFRBM	FRBM	SEM	p-value
DM	88.9	88.8	89.5	0.359	0.717
OM	93.3	93.4	93.8	0.416	0.890
GE	75.2^b^	75.3^b^	77.2^a^	0.305	0.006
CP	78.6^b^	80.6^b^	82.9^a^	0.617	<0.001
CF	48.4^b^	48.6^b^	52.6^a^	0.696	0.0130
NDF	66.8	65.6	67.2	0.689	0.633
ADF	80.6	79.5	79.2	0.441	0.420
EE	82.8^c^	87.4^b^	89.7^a^	0.559	<0.001
Crude ash	53.2^b^	56.2^a^	56.6^a^	0.442	0.001

1) n = 7/treatment.

a–c Means with different superscripts in the same row are significantly different (p<0.05).

CON, control group; UFRBM, unfermented rice bran meal group; FRBM, fermented rice bran meal group; SEM, standard error of the mean; DM, dry matter; OM, organic matter; GE, gross energy; CP, crude protein; CF, crude fiber; NDF, neutral detergent fiber; ADF, acid detergent fiber: EE, ether extract.

**Table 3:** Influences of incorporation of UFRBM or FRBM into rabbit’s diet on serum biochemical variables^1)^

Item	CON	UFRBM	FRBM	SEM	p-value
GLU (mmol/L)	7.93	7.86	8.33	0.233	0.696
TG (mmol/L)	0.700	0.766	0.800	0.0304	0.419
T. CHOL (mmol/L)	1.45	0.930	1.06	0.130	0.255
TP (g/L)	69.3	65.5	67.1	1.01	0.318
GLB (g/L)	42.3	39.8	40.9	0.816	0.469
ALB (g/L)	27.0	25.7	26.2	0.313	0.244
A: G ratio	0.640	0.648	0.645	0.0106	0.955
CREA (mcmol/L)	109	110	107	6.61	0.690
AST (U/L)	42.8	55.2	44.2	3.64	0.943
ALT (U/L)	50.6	45.6	51.1	6.84	0.333
AST: ALT ratio	0.648	0.601	0.693	0.0669	0.870
ALP (U/L)	115	112	109	14.0	0.774
BUN (mmol/L)	7.35	6.97	6.93	0.208	0.683

1) n = 7/treatment.

CON, control group; UFRBM, unfermented rice bran meal group; FRBM, fermented rice bran meal group; SEM, standard error of the mean; GLU, glucose; TG, triglycerides; T CHOL, total cholesterol; TP, total protein; GLB, globulin; ALB, albumin; CREA, creatinine; AST, aspartate aminotransferase; ALT, alanine aminotransferase; ALP, alkaline phosphatase; BUN, blood urea nitrogen.

**Table 4:** Influences of incorporation of UFRBM or FRBM into rabbit’s diet on antioxidant capacity indices and immunity status^1)^

Item	CON	UFRBM	FRBM	SEM	p-value
Antioxidant capacity indices					
T- AOC (mM)	0.449	0.538	0.502	0.0196	0.186
GSH-PX (U/mL)	88.1	83.2	114	7.25	0.158
SOD (U/mgprot)	65.4	69.3	66.1	1.44	0.533
CAT (U/mgprot)	9.19^ab^	8.85^b^	9.91^a^	0.174	0.035
MDA (nmol/mgprot)	4.14^a^	3.80^b^	4.07^a^	0.0576	0.026
Immunity status					
sIgA (ng/mL)	3.32	1.05	2.26	0.642	0.445

1) n = 7/treatment.

a,b Means with different superscripts in the same row are significantly different (p<0.05).

CON, control group; UFRBM, unfermented rice bran meal group; FRBM, fermented rice bran meal group; SEM, standard error of the mean; T-AOC, total antioxidant capacity; GSH-PX, glutathione peroxidase; SOD, superoxide dismutase; MDA, malondialdehyde activity; CAT, catalase activity; sIgA, secretory immunoglobulin A.

**Table 5:** Impacts of incorporation of UFRBM or FRBM into rabbit’s diet on the intestinal histomorphology^1)^

Item	CON	UFRBM	FRBM	SEM	p-value
Jejunal histomorphology					
Villus height (μm)	725^c^	744^b^	819^a^	10.2	<0.001
Crypt depth (μm)	106^b^	105^b^	111^a^	0.868	0.010
Villus height-to-crypt depth ratio	6.83^b^	7.05^b^	7.38^a^	0.0727	0.002
Cecal histomorphology					
Villus height (μm)	367^b^	374^b^	413^a^	6.45	0.001
Crypt depth (μm)	86.6	85.8	86.8	0.569	0.743
Villus height-to-crypt depth ratio	4.26^b^	4.39^b^	4.78^a^	0.0770	0.008

1) n = 7/treatment.

a–c Means with different superscripts in the same row are significantly different (p<0.05).

CON, control group; UFRBM, unfermented rice bran meal group; FRBM, fermented rice bran meal group; SEM, standard error of the mean.

**Table 6:** Effects of incorporation of UFRBM or FRBM into rabbit’s diet on digestive enzymes activity^1)^

Item	CON	UFRBM	FRBM	SEM	p-value
α-Amylase (U/mg protein)	5.43^ab^	4.30^b^	6.11^a^	0.290	0.024
Lactase (U/mg protein)	86.7	79.1	83.3	3.07	0.626
Lipase (U/mg protein)	52.4	59.9	60.9	2.51	0.338
Trypsin (U/Ml)	160^b^	178^ab^	181^a^	6.76	0.017
Chymotrypsin (U/mg protein)	6.65	8.73	9.41	0.554	0.099

1) n = 7/treatment.

a,b Means with different superscripts in the same row are significantly different (p<0.05).

CON, control group; UFRBM, unfermented rice bran meal group; FRBM, fermented rice bran meal group; SEM, standard error of the mean.

**Table 7:** Influences of incorporation of UFRBM or FRBM into rabbit’s diet on mRNA gene expression of nutrient transporters and tight junction^1)^

Item	CON	UFRBM	FRBM	SEM	p-value
Nutrient transporters					
*SLC3A1*	1.03^b^	1.77^a^	1.22^ab^	0.125	0.035
*SLC1A1*	1.05^b^	1.88^a^	1.27^b^	0.123	0.007
*SLC15A1*	1.07^b^	2.77^b^	5.51^a^	0.569	0.001
*SLC5A1*	1.06^b^	0.98^b^	1.93^a^	0.146	0.005
*SLC5A10*	0.833^a^	0.648^ab^	0.526^b^	0.0527	0.046
*FABP1*	1.26	1.67	1.72	0.189	0.575
Tight junctions					
*ZO-1*	1.02	0.919	1.03	0.130	0.806
*OCLN*	1.01^b^	1.38^a^	0.97^b^	0.0617	0.004
*CLDN1*	0.516	0.628	0.626	0.0726	0.823

1) n = 7/treatment.

a,b Means with different superscripts in the same row are significantly different (p<0.05).

CON, control group; UFRBM, unfermented rice bran meal group; FRBM, fermented rice bran meal group; SEM, standard error of the mean; *SLC3A1*, solute carrier family 3 member 1; *SLC1A1*, solute carrier family 1 member 1; *SLC15A1*, solute carrier family 15 member 1; *SLC5A1*, solute carrier family 5 member 1; *SLC5A10*, solute carrier family 5 member 10; *FABP1*, Fatty Acid Binding Protein 1; *ZO-1*, Zonula Occludens-1; *OCLN*, Occludin; *CLDN1*, Claudin 1.

**Table 8:** Effects of incorporation of UFRBM or FRBM into rabbit’s diet on α-diversity of cecum microbiota^1)^

Item	CON	UFRBM	FRBM	SEM	p-value
Observed species	1,493	1,491	1,463	13.0	0.619
Ace	1,813	1,775	1,763	14.2	0.355
Chao1	1,788	1,736	1,739	14.9	0.297
Shannon	5.71	5.78	5.65	0.0339	0.287
Simpson	0.989	0.991	0.987	0.0008	0.201

1) n = 7/treatment.

CON, control group; UFRBM, unfermented rice bran meal group; FRBM, fermented rice bran meal group; SEM, standard error of the mean.

**Table 9:** The relative abundance of top 30 bacterial community of rabbit’s cecum microbiota at genus level^1)^

Item	CON	UFRBM	FRBM	SEM	p-value
*Lachnospiraceae NK4A136 group*	7.57	8.71	8.77	0.516	0.592
*NK4A214 group*	7.62	7.85	8.08	0.504	0.939
*Christensenellaceae R-7 group*	7.15	4.49	5.33	0.560	0.138
*Ruminococcus*	4.17	4.14	5.40	0.356	0.273
*Bacteroides*	2.45	3.58	5.33	0.775	0.330
*Eubacterium siraeum group*	2.92	2.86	3.31	0.397	0.893
*Fusicatenibacter*	3.12	2.48	3.39	0.415	0.686
*dgA-11 gut group*	2.13	1.93	2.79	0.317	0.539
*Alistipes*	2.99	1.60	1.79	0.328	0.176
*Monoglobus*	1.80	1.83	1.73	0.121	0.947
*V9D2013 group*	0.549^b^	1.94^a^	0.892^ab^	0.241	0.036
*Subdoligranulum*	1.04	1.04	0.960	0.116	0.948
*Rikenellaceae RC9 gut group*	0.643	0.429	1.83	0.323	0.160
*Desulfovibrio*	1.06	0.687	1.02	0.113	0.350
*Rikenella*	0.402^b^	1.65^a^	0.510^b^	0.209	0.015
*28-4*	0.657	0.836	0.751	0.136	0.881
*Phascolarctobacterium*	0.814	0.549	0.526	0.161	0.748
*Marvinbryantia*	0.630	0.678	0.563	0.059	0.750
*Parabacteroides*	0.598	0.914	0.283	0.145	0.217
*Anaerofustis*	0.794	0.473	0.521	0.101	0.405
*Eubacterium Ruminantium group*	0.222	0.827	0.428	0.184	0.421
*Ruminiclostridium*	0.452	0.367	0.634	0.0524	0.099
*Parasutterella*	0.500	0.492	0.373	0.043	0.442
*Coprococcus*	0.452	0.546	0.254	0.102	0.524
*Anaerostipes*	0.228^ab^	0.892^a^	0.106^b^	0.129	0.017
*Incertae Sedis*	0.376	0.448	0.393	0.0383	0.751
*Candidatus Saccharimonas*	0.812	0.213	0.177	0.177	0.276
*Colidextribacter*	0.284	0.319	0.297	0.0374	0.936
*Akkermansia*	0.337	0.291	0.264	0.0448	0.818
*UCG-005*	0.294	0.215	0.340	0.0234	0.069

1) n = 7/treatment.

a,b Means with different superscripts in the same row are significantly different (p<0.05).

CON, control group; UFRBM, unfermented rice bran meal group; FRBM, fermented rice bran meal group; SEM, standard error of the mean.

**Table 10:** Relative abundance of metabolic pathways functions involved in carbohydrates metabolism at Kyoto Encyclopedia of Genes and Genomics level 3^1)^

Item	CON	UFRBM	FRBM	SEM	p-value
Amino sugar and nucleotide sugar metabolism	2.15	2.18	2.19	0.0090	0.329
Methane metabolism	2.09	2.06	2.02	0.0297	0.667
Pyruvate metabolism	1.82	1.83	1.81	0.0049	0.196
glycolysis-to-gluconeogenesis	1.67^b^	1.68^a^	1.68^a^	0.0024	0.008
Carbon fixation pathways in prokaryotes	1.65	1.63	1.63	0.0088	0.542
Starch and sucrose metabolism	1.51	1.54	1.57	0.0135	0.268
Pentose phosphate pathway	1.49	1.52	1.50	0.0082	0.346
Galactose metabolism	1.02	1.04	1.06	0.0115	0.431
Fructose and mannose metabolism	1.12	1.19	1.17	0.0119	0.082
Ascorbate and aldarate metabolism	0.11	0.11	0.11	0.0022	0.376
Citrate cycle (TCA cycle)	1.12	1.09	1.09	0.0095	0.360
Butanoate metabolism	0.966^ab^	0.995^a^	0.959^b^	0.0063	0.037
C5-Branched dibasic acid metabolism	0.464	0.454	0.455	0.0026	0.227
Carbohydrate digestion and absorption	0.0044^b^	0.0039^b^	0.0086^a^	0.0007	0.010
Glyoxylate and dicarboxylate metabolism	0.822	0.834	0.838	0.0042	0.321
Pentose and glucuronate interconversions	0.672	0.692	0.701	0.0080	0.349
Propanoate metabolism	0.909	0.933	0.908	0.0061	0.178
Phosphate metabolism	0.151	0.151	0.147	0.0013	0.343
Lipopolysaccharide biosynthesis	0.252	0.238	0.264	0.0074	0.383

1) n = 7/treatment.

a,b Means with different superscripts in the same row are significantly different (p<0.05).

CON, control group; UFRBM, unfermented rice bran meal group; FRBM, fermented rice bran meal group; SEM, standard error of the mean.

**Table 11:** Impacts of incorporation of UFRBM and FRBM into rabbit’s diet on cecum short chain fatty acids concentration^1)^

Item (Mm)	CON	UFRBM	FRBM	SEM	p-value
Total SCFA	33.1^b^	35.7^ab^	40.3^a^	1.15	0.038
Acetic acid	20.5^b^	24.9^ab^	27.8^a^	1.16	0.031
Propionic acid	4.84	5.30	6.16	0.241	0.078
Butyric acid	5.09^ab^	4.76^b^	6.33^a^	0.277	0.046
Isobutyric acid	0.639	0.791	0.711	0.0781	0.740
Valeric acid	0.431	0.333	0.444	0.0294	0.248
Isovaleric acid	0.256	0.432	0.320	0.0465	0.301

1) n = 7/treatment.

a,b Means with different superscripts in the same row are significantly different (p<0.05).

CON, control group; UFRBM, unfermented rice bran meal group; FRBM, fermented rice bran meal group; SEM, standard error of the mean; Total SCFA, total short chain fatty acid.

## SUPPLEMENTARY MATERIAL

**Supplement 1**. The ingredients and chemical composition of rabbit’s experimental diets (% on DM basis)

**Supplement 2**. The nutritional value of unfermented and fermented rice bran meal (% on DM basis)

**Supplement 3**. The jejunal primer sequences of targeted nutrient transporters and tight junction genes

**Supplement 4**. The functional predication categories for the metabolic pathways of rabbit’s cecum microbiota at KEGG level 1

**Supplement 5**. Relative abundance of metabolic pathways functions involved in rabbit’s cecum microbiota metabolism at KEGG level 2
