# Supplementary material for: Incorporation of unfermented or fermented de-oiled rice bran meal into a rabbit’s diet impacts growth performance, nutrient digestibility, cecal microbiota composition, and intestinal barrier function
Source: Anim Biosci. 2025 Apr 11;38(7):1459–74. doi: 10.5713/ab.24.0890 (PMC12229920; doi:10.5713/ab.24.0890)
Supplement: Supplementary file 1 [file ab-24-0890-Supplementary-1.pdf]

Supplement 1. The ingredients and chemical composition of rabbit's experimental diets (% on DM basis)

Ingredient	CON	UFRBM	FRBM
Corn grains	23.00	9.50	9.40
Soybean meal	10.30	7.50	7.50
Alfalfa hay	23.40	23.40	23.40
Peanut hay	25.00	20.90	21.00
Wheat bran	13.00	13.00	13.00
Rice bran meal	0.00	20.00	20.00
Soybean oil	2.00	3.00	3.00
NaCl	0.20	0.20	0.20
Cysteine	0.10	0.10	0.10
Methionine	0.10	0.10	0.10
Lysine	0.20	0.20	0.20
Calcium hydrogen	1.70	0.00	0.00
phosphate Mountain flower	0.00	1.10	1.10
Premix	1.00	1.00	1.00
Total (%)	100.00	100.00	100.00
Calculated chemical composition (%)			
DM	87.29	87.29	87.10
AME, MJ/kg	10.23	10.23	10.23
CP	15.60	15.65	15.63
CF	15.22	15.16	14.99
NDF	30.45	31.33	31.12
ADF	19.29	19.23	18.96
Lys	0.91	0.92	0.92
Cys+Met Ca	0.64	0.66	0.66
TP	0.86	0.85	0.85
	0.61	0.62	0.62
Detected chemical composition (%)			
DM	91.16	91.79	91.65
Moisture	8.84	8.21	8.35
Gross Energy, MJ/kg	18.31	18.47	18.62
C	15.92	16.10	16.78
P	4.82	5.33	5.71
EE	13.28	14.79	13.81
CE NDF	39.11	42.01	39.75
ADF	17.97	19.51	18.32
ADL	4.76	5.43	4.92
Hemicellulos	21.14	22.50	21.43
e Cellulose	13.21	14.08	13.40
Total ash AIA	9.17	9.98	9.28
	1.88	2.14	1.93

[1] CON, control group; UFRBM, unfermented rice bran meal group; FRBM, fermented rice bran meal group; DM, dry matter; AME, apparent metabolizable energy; CP, crude protein; EE, ether extract; CF, crude fiber; NDF, neutral detergent fiber; ADF, acid detergent fiber; ADL, acid detergent lignin; AIA, acid insoluble ash; Lys, lysine; Cys, cysteine; Met, methionine; Ca, calcium; TP, total phosphorus.

[2] Premix composition (per kilogram content), DM $\geq$ 88%; vitamin A, 260000-380000IU; vitamin D_3,40000-80000IU; vitamin E,$\geq$2500IU; copper, 440-1000mg ; zinc, 2000-4000mg; iron, 2000-4000mg; manganese, 1500-3000mg.
