# Supplementary material for: Incorporation of unfermented or fermented de-oiled rice bran meal into a rabbit’s diet impacts growth performance, nutrient digestibility, cecal microbiota composition, and intestinal barrier function
Source: Anim Biosci. 2025 Apr 11;38(7):1459–74. doi: 10.5713/ab.24.0890 (PMC12229920; doi:10.5713/ab.24.0890)
Supplement: Supplementary file 2 [file ab-24-0890-Supplementary-2.pdf]

Supplement 2. The nutritional value of unfermented and fermented rice bran meal (% on DM basis)

Detected chemical composition (%)	UFRBM	FRBM
DM	91.61	45.43
Moisture	8.39	54.57
Gross Energy, MJ/kg	17.10	17.59
CP	16.39	17.25
EE	1.57	1.93
CF	17.79	8.26
NDF	30.78	23.45
ADF	12.58	10.39
ADL	5.43	4.52
Hemicellulose	18.20	13.06
Cellulose	7.15	5.87
Total Ash	9.42	8.85
AIA	4.01	3.38

[1)] UFRBM, unfermented rice bran meal; FRBM, fermented rice bran meal; DM, dry matter; CP, crude protein; EE, ether extract; CF, crude fiber; NDF, neutral detergent fiber; ADF, acid detergent fiber; ADL, acid detergent lignin; AIA, acid insoluble ash.
