# Supplementary material for: Incorporation of unfermented or fermented de-oiled rice bran meal into a rabbit’s diet impacts growth performance, nutrient digestibility, cecal microbiota composition, and intestinal barrier function
Source: Anim Biosci. 2025 Apr 11;38(7):1459–74. doi: 10.5713/ab.24.0890 (PMC12229920; doi:10.5713/ab.24.0890)
Supplement: Supplementary file 3 [file ab-24-0890-Supplementary-3.pdf]

Supplement 3. The jejunal primer sequences of targeted nutrient transporters and tight junction genes

Gene	Accession number	Primers sequences (5'-3')	Product size (bp)
		Nutrient transporters	
SLC3A1	NM_001082242.1	F: GAGGAGCCCAACGCCAAGAAC<br>R: ACCACGACGAGCCAGAAGAGG	122
SLC1A1	NM_001082249.1	F: GTGATCGTGCTGAGTGCTGTG<br>R: CTTGGTGTCTGAGTCTTCGTTGTC	243
SLC15A1	NM_001082337.1	F: TTAGGCACCGCAGTAAGCAGTTTC<br>R: GGAACAGCACCCTCGTAACCATC	114
SLC5A1	NM_001101692.1	F: TGGTGGGCTCTGTGATCCTGAC<br>R: GGGAATGGCTCGCATGTACTTCTC	89
SLC5A10	NM_001082699.1	F: TGTTCACGCTGGGCATCTTC<br>R: CCACAGCGAAGTGCAGGTAG	186
FABP1	XM_002709637.4	F: GGGAAGGTCAAGACAGTGGT<br>R: CGCTTGAAGACAACGTCACC	137
		Tight junction	
ZO-1	XM_051822263.1	F: GCGGATGGTGCTACAAGTGATG<br>R: GCCTTCTGTATCTGTGTCTTCATAGTC	138
OCLN	XM_008262318.3	F: GGAGGACTGGATCAGGGAGT<br>R: GCCGCCATGTACTCTTCACT	192
CLDN1	NM_001089316.1	F: AAAGATGCGGATGGCTGTCA<br>R: GCCTGACCGAATTCATACCTTG	150
GAPDH	NM_001082253.1	F: CGCCTGGAGAAAGCTGCTAA<br>R: TTGAAGTCGCAGGAGACGAC	119

[1)] *SLC3A1*, solute carrier family 3 member 1; *SLC1A1*, solute carrier family 1 member 1; *SLC15A1*, solute carrier family 15 member 1; *SLC5A1*, solute carrier family 5 member 1; *SLC5A10*, solute carrier family 5 member 10; *FABP1*, Fatty Acid Binding Protein 1; *ZO-1*, Zonula Occludens- 1; *OCLN*, Occludin; *CLDN1*, Claudin 1; *GAPDH*, Glyceraldehyde Phosphate dehydrogenase (moonlighting reference gene).
