# Supplementary material for: Incorporation of unfermented or fermented de-oiled rice bran meal into a rabbit’s diet impacts growth performance, nutrient digestibility, cecal microbiota composition, and intestinal barrier function
Source: Anim Biosci. 2025 Apr 11;38(7):1459–74. doi: 10.5713/ab.24.0890 (PMC12229920; doi:10.5713/ab.24.0890)
Supplement: Supplementary file 4 [file ab-24-0890-Supplementary-4.pdf]

Supplement 4. The functional predication categories for the metabolic pathways of rabbit's cecum microbiota at KEGG level 1

Item	CON	UFRBM	FRBM	SEM	p-value
Cellular processes	4.43	4.68	4.80	0.176	0.708
Environmental information processing	8.78	9.75	10.29	0.432	0.374
Genetic information processing	6.46	6.25	6.16	0.138	0.687
Human diseases	2.59	2.56	2.50	0.0474	0.780
Metabolism	76.6	75.6	75.1	0.525	0.527
Organismal systems	1.06	1.10	1.05	0.0377	0.847

[1)] SEM: standard error of the mean.

[2)] [a-c] Means with different superscripts in the same row are significantly different ($p<0.05$).

[3)] CON, control group; UFRBM, unfermented rice bran meal group; FRBM, fermented rice bran meal group; (n = 7/treatment).
