# Supplementary material for: Incorporation of unfermented or fermented de-oiled rice bran meal into a rabbit’s diet impacts growth performance, nutrient digestibility, cecal microbiota composition, and intestinal barrier function
Source: Anim Biosci. 2025 Apr 11;38(7):1459–74. doi: 10.5713/ab.24.0890 (PMC12229920; doi:10.5713/ab.24.0890)
Supplement: Supplementary file 5 [file ab-24-0890-Supplementary-5.pdf]

**Supplement 5.** Relative abundance of metabolic pathways functions involved in rabbit's cecum microbiota metabolism at KEGG level 2

| Item | CON | UFRBM | FRBM | SEM | p-value |
|---|---|---|---|---|---|
| Carbohydrate metabolism | 10.0 | 10.4 | 10.3 | 0.125 | 0.438 |
| Amino acid metabolism | 6.60 | 6.45 | 6.45 | 0.0621 | 0.557 |
| Membrane transport | 5.12 | 5.87 | 5.33 | 0.276 | 0.203 |
| Energy metabolism | 4.84 | 4.66 | 4.61 | 0.136 | 0.798 |
| Signal transduction | 3.65 | 3.88 | 3.95 | 0.198 | 0.831 |
| Metabolism of cofactors and | 3.55 | 3.43 | 3.38 | 0.0535 | 0.423 |
| vitamins Nucleotide metabolism | 3.08 | 3.00 | 2.97 | 0.0458 | 0.668 |
| Translation | 2.94 | 2.78 | 2.72 | 0.102 | 0.713 |
| Replication and repair | 2.02 | 2.02 | 2.00 | 0.0201 | 0.951 |
| Lipid metabolism | 1.82 | 1.86 | 1.82 | 0.0461 | 0.955 |
| Biosynthesis of other secondary metabolites | 1.31 | 1.32 | 1.31 | 0.0137 | 0.625 |
| Glycan biosynthesis and metabolism | 1.30 | 1.29 | 1.18 | 0.0583 | 0.645 |
| Xenobiotics biodegradation and metabolism | 1.14 | 1.12 | 1.21 | 0.0252 | 0.357 |
| Folding, sorting and degradation | 1.18 | 1.14 | 1.13 | 0.0144 | 0.392 |
| Metabolism of other amino acids | 1.15 | 1.14 | 1.14 | 0.0062 | 0.893 |
| Metabolism of terpenoids and polyketides | 0.826 | 0.810 | 0.826 | 0.0486 | 0.939 |
| Transcription | 0.322 | 0.298 | 0.296 | 0.0214 | 0.875 |
| Transport and catabolism | 0.307 | 0.293 | 0.268 | 0.0095 | 0.256 |

1) SEM: standard error of the mean.

2) a-c Means with different superscripts in the same row are significantly different ($p<0.05$).

3) CON, control group; UFRBM, unfermented rice bran meal group; FRBM, fermented rice bran meal group; (n = 7/treatment).
